# Research Cooperative Groups in Pediatric Palliative Care Research

**DOI:** 10.1089/pmr.2020.0043

**Published:** 2020-12-14

**Authors:** Terrah Foster Akard, Mary Jo Gilmer

**Affiliations:** School of Nursing, Vanderbilt University, Nashville, Tennessee, USA.

**Keywords:** hospice and palliative care nursing, interdisciplinary research, palliative care, palliative medicine, research

## Abstract

Research cooperative groups aim to facilitate collaborative and rigorous palliative care research. The purpose of this article is to (1) demonstrate how cooperative groups are taking formal and sustainable steps with commitment to pediatric palliative care research programs and (2) provide an example of how one cooperative group is implementing these innovative efforts to partner with programs to integrate pediatrics on an expanding scale. Details are described for how pediatric studies can benefit from cooperative group infrastructure and expertise. In turn, we describe how cooperative groups can benefit from collaborating on pediatric studies through broadening of data dictionaries, data repositories, and reach in palliative care research communities.

## Introduction

Substantial progress has been made for the past 20 years in the care of children with serious illnesses. However, crucial gaps still exist in the evidence to inform our understanding of the struggles families face and how to address the needs of pediatric palliative care patients and their families. A potential limitation of current pediatric palliative care research is that a lack of research infrastructure may contribute to studies being conducted that are not well suited to answer the research questions they seek to address. One strategy to improve pediatric palliative care research infrastructure is through use of cooperative organized structures, such as the Pediatric Palliative Care Research Network (PPCRN)^1^ and Palliative Care Research Cooperative (PCRC) group. The purpose of this special report is to highlight benefits of cooperative research, which enable the field of pediatric palliative care to flourish. Specifically, this article (1) demonstrates how cooperative groups are taking formal and sustainable steps with commitment to pediatric palliative care research programs and (2) provide an example of how such groups are implementing these innovative efforts to partner with programs to integrate pediatrics on an expanding scale.

Two examples of well-established research cooperative groups are the PPCRN and PCRC. The PPCRN was established in 2006, is focused on fostering a community of pediatric palliative care investigators and strengthening the pipeline of young and early career investigators, and currently includes 118 members.^1^ The PCRC was established in 2010 to facilitate collaborative and rigorous palliative care research.^2,3^ Of 613 PCRC members, 92 (15%) have indicated a pediatric focus. Although such PCRCs are now well established, a better understanding for how such groups can benefit pediatric palliative care research is needed. Tables 1 and 2 present case examples from a pediatric palliative care study^4–7^ conducted in collaboration with the PCRC. Table 1 illustrates how cooperative groups can benefit pediatric palliative care research as demonstrated by PCRC support domains, potential benefits to researchers, and examples from our study. Conversely, Table 2 illustrates how pediatric palliative care research can benefit cooperative groups as demonstrated by research domains, potential benefits to cooperative groups, and examples from our study. These exemplars show how cooperative group resources can be utilized in pediatric palliative care research. Contributions to and from cooperative groups are described in the following sections.

**Table 1.** How Cooperative Groups Can Benefit Pediatric Palliative Care Research

PCRC support domain	Potential benefit to researchers	Example from our study
Data harmonization	Standardized datasets across studies and increased efficiency	PCRC common data elements facilitated execution of the development of our study's REDCap database
Participant registry	Documents all PCRC study participants, providing an account of overall PCRC enrollment and activity	Our study deposited data into the PCRC participant registry
Quality assurance	Improves overall research quality through assessment for data completeness and assurance that NIH requirements are met to support appropriate participant tracking	Audits reviewed participant enrollment, retention, and missing data, which helped ensure our analysis sample was appropriate to achieve study aims
Caregiver expertise	Caregiver Core offers research expertise related to caregiver-specific issues	Provided caregiver expertise to consider for articles, including secondary analyses mid-study, and journal selections for dissemination, and guidance for caregiver recruitment strategies
Measurement expertise	Expertise provided to help guide selection of measures based on sound psychometrics and established feasibility with target population	The PI consulted with the Measurement Core during study design to identify optimal measures and further enhance the electronic data collection method proposed in the study (e.g., additional strategies to decrease missing data, methods for participant reminders)
Study closeout	Support to close out the study database and prepare it to meet the PCRC data sharing requirements	Shared study database with the PCRC, allowing the PCRC to coordinate future data sharing requests and increase availability of our dataset to inform future studies

NIH, National Institutes of Health; PCRC, Palliative Care Research Cooperative group; PI, principal investigator; PPCRN, Pediatric Palliative Care Research Network.

**Table 2.** How Pediatric Palliative Care Research Can Benefit Cooperative Groups

Research domain	Potential benefit to cooperative group	Example from our study
Pediatric data harmonization	Data elements specific to pediatrics	Added parent-proxy child, disease, and palliative care demographics to standardized data elements
Pediatric measures	Contribute pediatric self-report and parent proxy report measures to cooperative group resources	Added pediatric measures to the PCRC instrument database
Pediatric data sharing	Data for pediatric secondary analyses, pilot research projects, and aggregated analyses	Study database and data dictionary deposited into the PCRC data repository
Increased visibility	Increased cooperative group and pediatric palliative care research visibility	PI has established strong collaborations with the PCRC and its members since the beginning of the study, leading to invited presentations at the annual investigator meeting and leadership positions within the organization

## Cooperative Group Contributions to Research

Cooperative groups can play a significant role in supporting pediatric palliative care research. For example, the PCRC resources include data harmonization, participant registry, quality assurance, caregiver research expertise, measurement expertise, and study closeout support. Each of these domains of PCRC support and potential benefits to researchers will be described with specific examples from our study in Table 1.

### Data harmonization

The PCRC uses common data elements, measurement instruments, and data dictionaries to standardize datasets to support data harmonization across studies and increase efficiency. The PCRC also houses a de-identified data repository and solicits investigators to deposit their data for other researchers to ask important research questions and conduct secondary data analyses.

### Participant registry

The PCRC study participant registry documents all participants enrolled in PCRC studies, providing an accounting of overall PCRC enrollment and activity. The PCRC developed and maintains this trans-PCRC participant registry.

### Quality assurance

PCRC statisticians conduct annual quality assurance reviews of study databases to ensure compliance with PCRC standards. Quality assurance includes assessment to ensure that PCRC data standards are followed (including alignment with the predefined data dictionary). Reviews also provide study closeout information for researchers to plan ahead for materials required to be submitted at study end.

### Caregiver expertise

The PCRC Caregiver Research Core ensures that studies align with state-of-the-art caregiver research methods.

### Measurement expertise

The PCRC Measurement Core assists researchers to ensure that instruments align with PCRC standards.

### Study closeout support

At study end, the PCRC lead statistician and informatics team worked with researchers to close out the study database and prepare it to meet the PCRC data sharing requirements. This allows for the PCRC to coordinate any future data sharing requests from other researchers and increases availability of our dataset to inform future studies.

These cooperative group domains can provide support and quality control and assist with successful completion and increased rigor of pediatric palliative care research. While accomplishing their mission, research cooperative groups can support researchers in successfully addressing the aims of research studies and expanding the knowledge base in pediatric palliative care.

## Research Contributions to Cooperative Groups

The contribution of pediatric palliative care researchers can substantially expand and pragmatically benefit research cooperative group paradigms. Pediatric-specific contributions in areas of data harmonization, measures, data sharing, and visibility within cooperative groups and beyond can be added as a result of pediatric palliative care research. These contribution domains are described hereunder and summarized with specific examples from our study in Table 2.

### Pediatric data harmonization

Studies can contribute data elements specific to pediatric parent-proxy report and pediatric palliative care to cooperative groups. Researchers can work with collaborative groups to ensure that these important categories of data are part of the collaborative information ecosystem.

### Pediatric-related measures

Pediatric-specific measures can be added to cooperative group resources, specifically for child self-report and parent caregivers of pediatric patients, as the majority of current family caregiver measures are adult based. In this way, researchers can both draw from and contribute to the palliative care community.

### Pediatric-related data sharing

In addition to data availability in accordance with National Institutes of Health (NIH) requirements, research databases and data dictionaries can be invested into cooperative group repositories of shared data to support subsequent secondary analyses, pilot research projects, and aggregated analyses.

### Increased cooperative group and pediatric palliative care research visibility

Pediatric palliative care research studies can contribute to increased visibility of pediatric expertise of research cooperative groups and their members. Study dissemination of results to peer-reviewed journals and national conferences include acknowledgement of collaborating cooperative groups, increasing their visibility and exposing the benefits of cooperative groups to pediatric palliative care research across the nation. Collaboration between research and cooperative groups can also provide leadership opportunities for pediatric researchers within the cooperative group community. Such increased research visibility can help grow the network of pediatric palliative care researchers by encouraging new members across disciplines and levels (e.g., doctoral students and senior faculty researchers) to join cooperative groups, thus helping to support established pediatric palliative care researchers and grow the pipeline of junior investigators in palliative care.

## Discussion

Examples from our project demonstrate how cooperative groups can offer bidirectional benefits from recruitment through dissemination and demonstrate that their commitment to pediatric palliative care research is wide, far-reaching, and growing. With a variety of interacting resources, benefits of cooperative groups include assistance for both novice and expert researchers. Research design recommendations and pediatric measures stored in instrument libraries can be used by pediatric palliative care researchers with confidence, knowing they have been endorsed through published systematic reviews. Implications for additional expansion of use of the assistance offered by existing palliative care cooperative groups could include (1) increase in pediatric palliative care researcher involvement and (2) replication of collaborative models for use in other settings and conditions (e.g., university, national, and international levels). Each of these opportunities is described further as follows.

An important implication for future study is the addition of more pediatric palliative care researchers to be actively involved in existing palliative care collaborative groups. Based in children's hospitals and communities across the country, researchers who concentrate on building the knowledge base and evidence-based practice with children and families can benefit from scientific collaboration and advanced knowledge. This may be accomplished most efficaciously through membership in research group cooperatives where resources and expertise are shared. The addition of pediatric expertise is one of the goals of the PCRC's U2C, and as such, the PCRC added leading pediatric palliative care researchers to the Steering Committee and Scientific Review Committee and have regular meetings with pediatric palliative care research leaders. Thus, both the PPCRN and PCRC are strongly committed to continuing and further expanding their support for pediatric palliative care research.

The collaborative model of palliative care cooperative groups is one worthy of replication and expansion. For example, similar models could be used at the university level to facilitate research collaboration within institutions. Other researchers could use components of palliative care cooperative group models to develop national cooperative research groups for nonpalliative care research areas of interest. Internationally, these models could be used to engage more global pediatric palliative care researchers into our existing palliative care groups to better address the needs of researchers working to enhance the quality of lives of some of the 21.5 million children in other countries and settings. With an emphasis on scientifically based methods, results provide meaningful evidence that may be useful across settings, borders, and languages.

## Conclusion

Our study was pioneering in its use of formalized collaborative resources and infrastructure for *pediatric* palliative care research. Palliative care researchers can greatly benefit from the value of a collaborative group, including enhanced family caregiver research, measurement and study design, and data harmonization. In addition, studies can contribute back to a cooperative group's mission through the development of new resources related to a specific population (e.g., pediatrics), including measures, methods, and standards for palliative care research. Although the PPCRN is pediatric-specific and similar in its mission of fostering multicentered collaborative interdisciplinary research, our study was one of the first to demonstrate the value of the PCRC related to pediatrics and supports long-term sustainability. Pediatric palliative care researchers can benefit from joining a collaborative research group to further strengthen our pediatric community.

## Abbreviations Used

NIH: National Institutes of Health
PCRC: Palliative Care Research Cooperative group
PI: principal investigator
PPCRN: Pediatric Palliative Care Research Network
